# Effect of climate change in lizards of the genus *Xenosaurus* (Xenosauridae) based on projected changes in climatic suitability and climatic niche conservatism

**DOI:** 10.1002/ece3.4200

**Published:** 2018-06-25

**Authors:** Christian Berriozabal‐Islas, João Fabrício Mota Rodrigues, Aurelio Ramírez‐Bautista, Jorge L. Becerra‐López, Adrián Nieto‐Montes de Oca

**Affiliations:** ^1^ Ecología de Poblaciones Centro de Investigaciones Biológicas Universidad Autónoma del Estado de Hidalgo Ciudad Universitaria (Ciudad del Conocimiento) Hidalgo México; ^2^ Departamento de Ecologia Universidade Federal de Goiás Goiânia Brazil; ^3^ Laboratorio de Cambio Climático y Conservación de Recursos Naturales Centro de Estudios Ecológicos Facultad de Ciencias Biológicas Universidad Juárez del Estado de Durango Gomez Palacio México; ^4^ Museo de Zoología Departamento de Biología Evolutiva Facultad de Ciencias Universidad Nacional Autónoma de México Ciudad de México México

**Keywords:** climatic variables, cloud forest, conservation, endemism, extinction, niche overlap, tropical, vulnerability

## Abstract

Accelerated climate change represents a major threat to the health of the planet's biodiversity. Particularly, lizards of the genus *Xenosaurus* might be negatively affected by this phenomenon because several of its species have restricted distributions, low vagility, and preference for low temperatures. No study, however, has examined the climatic niche of the species of this genus and how their distribution might be influenced by different climate change scenarios. In this project, we used a maximum entropy approach to model the climatic niche of 10 species of the genus *Xenosaurus* under present and future suitable habitat, considering a climatic niche conservatism context. Therefore, we performed a similarity analysis of the climatic niche between each species of the genus *Xenosaurus*. Our results suggest that a substantial decrease in suitable habitat for all species will occur by 2070. Among the most affected species, *X*. *tzacualtipantecus* will not have suitable conditions according to its climatic niche requirements and *X*. *phalaroanthereon* will lose 85.75% of its current suitable area. On the other hand, we found low values of conservatism of the climatic niche among species. Given the limited capacity of dispersion and the habitat specificity of these lizards, it seems unlikely that fast changes would occur in the distribution of these species facing climate change. The low conservatism in climatic niche we found in *Xenosaurus* suggests that these species might have the capacity to adapt to the new environmental conditions originated by climate change.

## 1 INTRODUCTION

The climatic niche of a species is the set of climatic variables delimiting the necessary conditions for it to reproduce and survive (Bonetti & Wiens, 2014), which is determined by adaptation to its environment (Zúñiga‐Vega et al., 2017) and the legacy of ancestry (Ackerly & Reich, 1999). Considering this, studying the specialization to a limited set of climatic conditions of a species could be important for understanding its response to climate change (Deutsch et al., 2008). The changes in climate originated by anthropogenic activities can have serious potential consequences for most species (Albon et al., 2017), particularly those that strictly depend on specific conditions of habitat (Parmesan, 2006).

In this context, Wiens et al. (2010) and Liu et al. (2017) proposed that the threat of climate change can be analyzed by climatic niche conservatism. This concept refers to the retention of certain characteristics of the ancestral fundamental niche over time and space (Wiens et al., 2010). Accordingly, it has been suggested that if the climatic tolerance of a species is not extensive enough to face new environmental conditions, then those species with strong niche conservatism must migrate or become extinct (Jackson, Gergel, & Martin, 2015; Wiens et al., 2010). For example, some reports have shown that several lizard species tend to maintain similar thermal preferences despite inhabiting different types of environments (Andrews, 1998; Bogert, 1949; Díaz de la Vega‐Pérez, Jiménez‐Arcos, Manríquez‐Morán, & Méndez‐De la Cruz, 2013; Grigg & Buckley, 2013; Rocha & Vrcibradic, 1996; Schall, 1977). This represents strong evolutionary evidence of the presence of conservatism in optimal thermal preferences in these groups of vertebrates (Adolph, 1990; Menezes & Rocha, 2011; Pianka, 1970; Stevens, 1982; Winne & Keck, 2004).

Range‐restricted species often are particularly vulnerable to extinction due to the climate change that is happening presently; therefore, endemic species could be strongly affected (Ballesteros‐Barrera, Martínez‐Meyer, & Gadsden, 2007; Malcolm, Liu, Neilson, Hansen, & Hannah, 2006). This condition could be even more problematic if these species tend to conserve their climatic niche (Wiens et al., 2010). Many studies have used species distribution modeling to predict habitat suitability of endemic species in the future (García, Ortega‐Huerta, & Martínez‐Meyer, 2013; Thuiller et al., 2006). However, evaluating whether these studied species conserve their niche has not been performed commonly. Therefore, besides evaluating the availability of areas with suitable climatic conditions for species in the future, it is also important to evaluate whether species show a tendency to conserve their climatic niche, which would help provide more information to better understand their capacity of response to climate change; these data could be obtained from reptiles, because as ectothermic organisms, they are good models to use to assess the impacts of climate change (Huey & Kingsolver, 1993; Valenzuela‐Ceballos, Castañeda‐Gaytán, Rioja‐Paradela, Carrillo‐Reyes, & Bastiaans, 2015).

**Figure 1:**
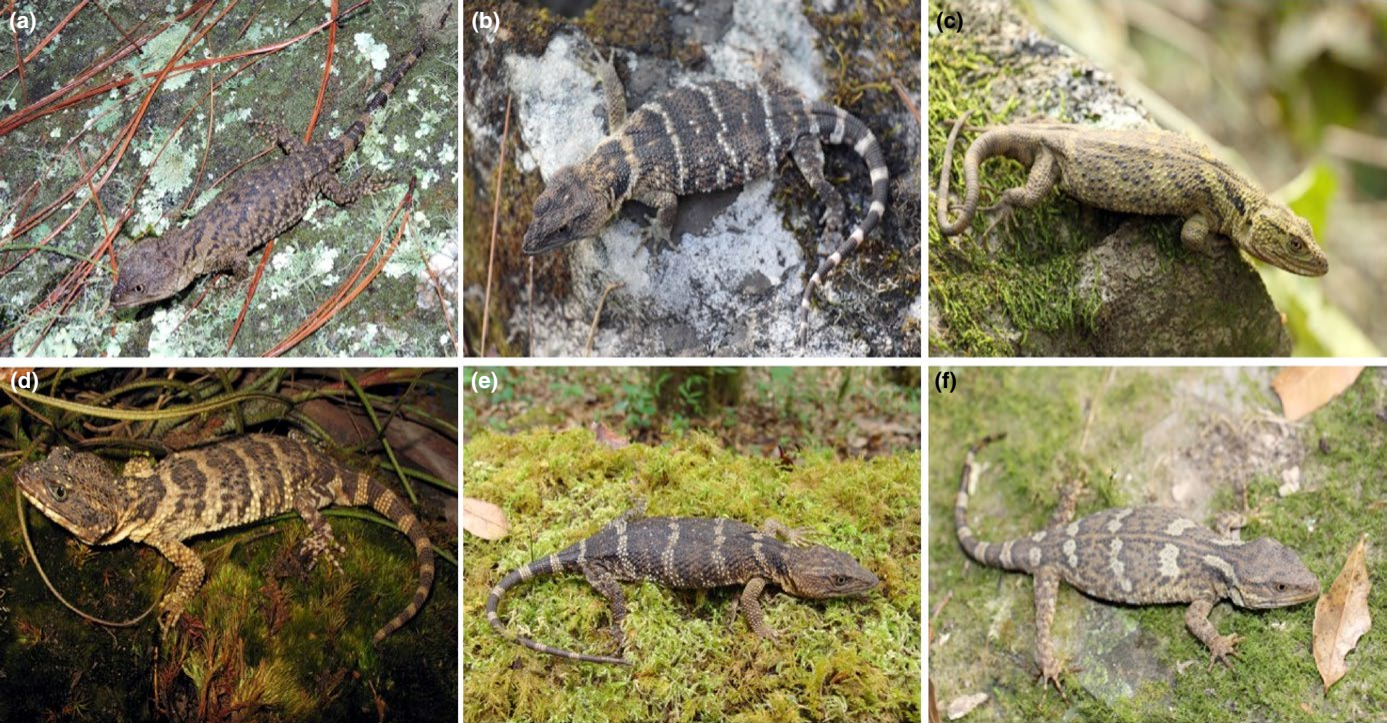
Some species of *Xenosaurus* included in this study (a) *Xenosaurus agrenon*, (b) *X. mendozai*, (c) *X. newmanorum*, (d) *X. phalaroanthereon*, (e) *X.  platyceps*, and (f) *X. tzacualtipantecus*

Lizards of the genus *Xenosaurus* comprise a group of northern forms of Laurasian origin with a diversification in North America; therefore, they are considered as one of the oldest groups of lizards (Macey et al., 1999; Bhullar, 2011; Figure 1). Currently, distribution of the *Xenosaurus* species is associated with mountain chains that occur from northeastern Mexico to Alta Verapaz, Guatemala (Bhullar, 2011). Species of *Xenosaurus* occur in a broad altitudinal range of approximately 500–2,360 m, and they can be found in a wide variety of habitats, ranging from xerophytic tropical scrub to tropical montane cloud forest, and tropical rain forest (King & Thompson, 1968). Most species of the genus share behavioral and physiological characteristics, such as being thermoconformists and thigmotherms with preferences for relatively low temperatures (García‐Rico, Díaz de la Vega‐Pérez, Smith, Lemos‐Espinal, & Woolrich‐Piña, 2015). Some authors have suggested that the entire genus *Xenosaurus* might be very susceptible to rapid environmental changes (Nieto‐Montes de Oca, 1999; Sinervo et al., 2010), which could be supported by the fact that most of these species are microendemic and have restricted dispersal abilities (Zamora‐Abrego & Ortega‐León, 2016). No study, however, has properly evaluated how climate changes might influence this group of lizards; therefore, the following question becomes relevant: how would lizard species of *Xenosaurus* respond if the conditions of their climatic niche are modified because of climate change?

In this project, we evaluated: (a) the extent of areas with suitable climatic conditions for the occurrence of the *Xenosaurus* species in the near future (2070), considering that these lizards conserve their climatic niche; and (b) whether the various species of *Xenosaurus* have different climatic niches, which could be evidence of lack of niche conservatism, favoring taxa with reduced areas with suitable living conditions in the future.

## 2 METHODS

### 2.1 Taxon sampling

**Figure 2:**
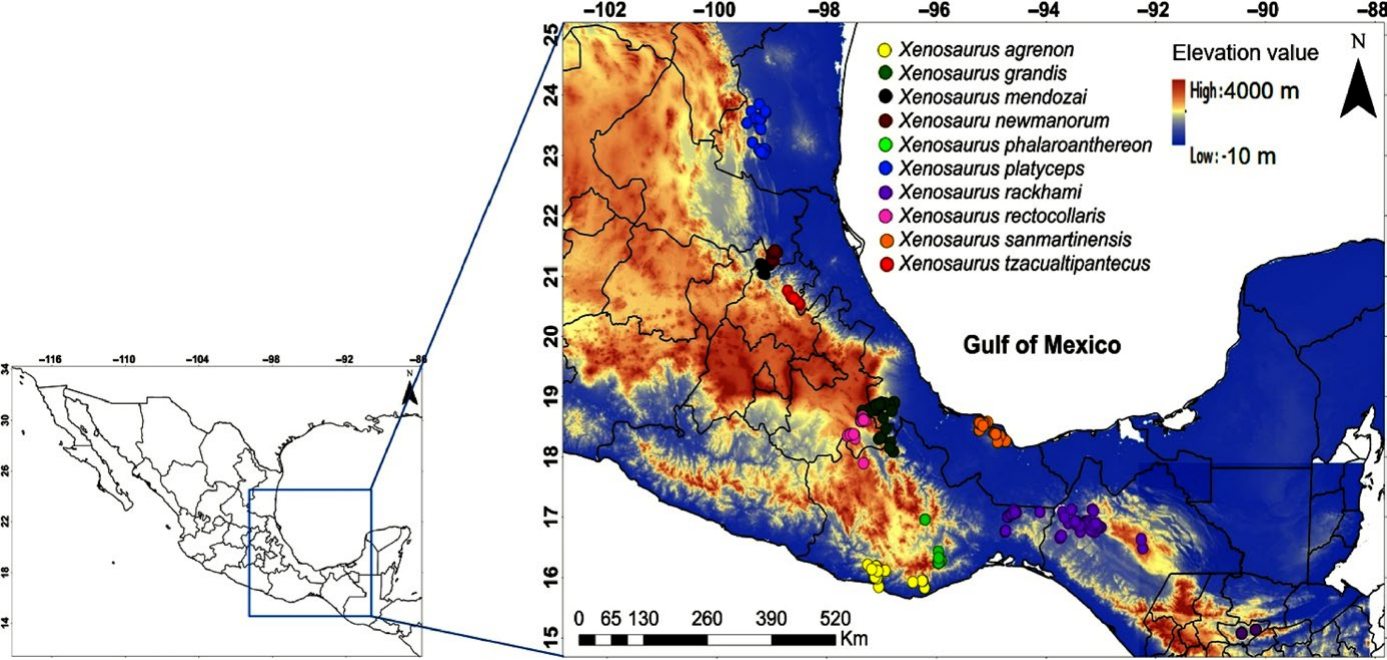
Distribution of the records of the 10 species of *Xenosaurus*. Map showing the records evaluated in our study

The genus *Xenosaurus* is composed of 12 described species: *Xenosaurus agrenon*,* X*. *arboreus*,* X*. *grandis*,* X*. *mendozai*,* X*. *newmanorum*,* X*. *penai*,* X*. *phalaroanthereon*,* X*. *platyceps*,* X*. *rackhami*,* X*. *rectocollaris*,* X*. *sanmartinensis*, and *X*. *tzacualtipantecus* (Nieto‐Montes de Oca, García‐Vázquez, Zúñiga‐Vega, & Schmidt‐Ballardo, 2013). Additionally, six more species were recognized but not described by Nieto‐Montes de Oca et al. (2017). We included 10 species of *Xenosaurus* in this study, following the taxonomy proposed by Nieto‐Montes de Oca et al. (2017) in a phylogenetic analysis of the genus based on RADseq data: *X*. *agrenon*,* X*. *grandis*,* X*. *mendozai*,* X*. *newmanorum*,* X*. *phalaroanthereon*,* X*. *platyceps*,* X*. *rackhami*,* X*. *rectocollaris*,* X*. *sanmartinensis*, and *X*. *tzacualtipantecus*. The species *X*. *arboreus*,* X*. *penai*, and the other six species mentioned but not described by Nieto‐Montes de Oca et al. (2017) were not included in this analysis because available information is scarce. Occurrence data of the *Xenosaurus* species were obtained from three sources: (a) field geographic records collected by the authors in a period of more than 10 years, since 2006, (b) published records (Ballinger, Lemos‐Espinal, Sanoja‐Sarabia, & Coady, 1995; Ballinger, Lemos‐Espinal, & Smith, 2000; García‐Rico et al., 2015; Lemos‐Espinal & Smith, 2005; Lemos‐Espinal, Smith, & Ballinger, 2003a,b; Nieto‐Montes de Oca, 1999; Rojas‐González, Jones, Zúñiga‐Vega, & Lemos‐Espinal, 2008; Woolrich‐Piña, Lemos‐Espinal, Oliver‐López, & Smith, 2012; Zamora‐Abrego & Ortega‐León, 2016), and (c) unpublished data of ANMO. The number of occurrence records collected for each species was *X*. *agrenon* (25), *X*. *grandis* (110), *X*. *mendozai* (60), *X*. *newmanorum* (40), *X*. *phalaroanthereon* (12), *X*. *platyceps* (62), *X*. *rackhami* (70), *X*. *rectocollaris* (31), *X*. *sanmartinensis* (41), and *X*. *tzacualtipantecus* (40). The database for this study consisted of 491 geographic records (Figure 2).

Specimen sampling was performed on public and private lands, with the corresponding permission of the owners. Fieldwork was carried out under the scientific collecting permit SGPA/DGVS/02419/13, granted by the Mexican Secretary for Environment and Natural Resources (SEMARNAT). We only obtained records based on observation and invasive procedures were not performed on the lizards.

### 2.2 Selection of climatic variables

Climatic information was obtained from the 19 current climatic layers available in WorldClim (Hijmans, Cameron, Parra, Jones, & Jarvis, 2005). These were used to describe the climatic niche of each *Xenosaurus* species. These climatic layers contain annual means of the meteorological conditions recorded from the period 1950–2000 with a spatial resolution of 2.5 arc‐minutes. We trimmed the spatial extent of the variables in ArcGIS to include the area from Mexico and Guatemala and included the resolution of our data in one km^2^ (ESRI 2014).

Subsequently, to identify the variables with the greatest contribution in explaining the spatial‐environmental variation for each species, an analysis of bivariate correlation of Pearson was performed among all the environmental variables pairs to check for collinearity among the input variables in the program STATISTICA ver 10 (StatSoft Inc, 2004; Merow, Smith, & Silander, 2013; Varela, Anderson, García‐Valdés, & Fernández‐González, 2014), and only variables with correlation values of less than 0.7 (*r* < 0.7) were retained. Seven climatic variables were selected when performing this procedure: Bio1 (annual mean temperature), bio7 (annual range temperature), bio11 (annual mean temperature of the coldest quarter), bio12 (annual precipitation), bio 14 (precipitation of the driest month), bio17 (precipitation of the driest quarter), and bio18 (precipitation of the warmest quarter).

### 2.3 Climatic niche modeling (habitat suitability)

We used the Maximum Entropy modeling method (Phillips, Anderson, & Schapire, 2006) implemented in the software MaxEnt version 3.3.1 (Phillips, Dudik, & Schapire, 2004), to develop niche models of habitat suitability for each *Xenosaurus* species. MaxEnt is a maximum entropy‐based program that estimates the probability distributions of a species in a geographic space using occurrence records and environmental data (Phillips et al., 2006). This modeling algorithm only requires presence data and has relatively good performance when compared to other presence‐only methods (Elith et al., 2006). To establish the areas that maintain the environmental conditions of the species and that could be occupied by them (Burgman & Fox, 2003; Elith et al., 2011), MaxEnt generates habitat suitability maps scaling from zero (low suitability) to 1 (high suitability; Elith et al., 2011). Data of the seven bioclimatic variables used for the study were collected for the whole background using the software ArcMap version 10.3.

Successful calibration is important for datasets that suffer from sampling bias and for studies that require transfer models through space or time, for example, climate change response (Elith, Kearney, & Phillips, 2010; Moreno‐Amat et al., 2015). Therefore, for the construction of the models, the dataset used in MaxEnt was randomly divided as follows: 75% of the data was used for the model construction or calibration, and the remaining 25% was used to evaluate the adjustment of the model (Cianfrani, Lay, Hirzel, & Loy, 2010).

Information obtained from each model was projected in order to identify potential areas under current climatic conditions and the scenario of the climate change RCP85‐2070. We used projections to the year 2070 employing the environmental variables records available on the WorldClim database, which are calculated from future climate projections of General Circulation Models (Hijmans et al., 2005). The RCP 85 assumes that global greenhouse gas concentration trajectories will continue to rise throughout the XXI century and will stabilize in the year 2100 (Meinshausen et al., 2011). We evaluated MaxEnt model performance using a fifteen‐fold cross‐validation of the area under the curve (AUC) of the receiver operating characteristic curve (ROC). Models with an AUC score of 0.5 indicated a model performing randomly, while models with an AUC score of 1 indicated a perfect model (Lobo, Jiménez‐Valverde, & Real, 2007; Phillips, Dudik, & Schapire, 2004). The best model among the 15 cross‐validations for each species (the model with the highest AUC) was converted into a binary map (presence‐absence), using the logistic threshold considering maximum training sensitivity plus specificity, which had the best characteristics for each model. These conversions were performed in ArcMap version 10.3. Finally, to assess the impact of climate change in *Xenosaurus*, the percentage of change between current and future conditions was obtained using the formula: % of change = [(S1–S0)/S0]*100%, where S0 is the suitable area for each species according to the baseline scenario and S1 is the suitable area for each species under future climatic conditions (Gutiérrez & Trejo, 2014).

### 2.4 Similarity of climatic niche

Comparison of climatic niche among the 10 species of the genus was carried out using the analytical framework developed by Broennimann et al. (2012) available in the library ecospat (Di Cola et al., 2017) for R version 3.3.2 R Core Team, including the environmental variables retained in the analyses of bivariate correlation. We used the Principal Component Analysis Approach (PCA‐environmental), where we extracted the first two axes of a PCA including the seven bioclimatic variables selected in our study to represent the climatic niche. Environmental space was divided in a grid of 100 × 100 cells, and each cell represented a single vector of the environmental conditions that occur in one or more sites in the geographic space (Broennimann et al., 2015; Hu et al., 2016). A Kernel density function was employed to calculate the density of occurrence of each species and number of sites, with particular environmental conditions for each cell of the environmental space.

Niche overlap between species in the environmental space was measured by the Schoener D metric (Schoener, 1970), and niche similarity tests were performed according to Warren, Glor, and Turelli (2008), using 100 randomizations in the null model. When the observed niche overlap value was significant (*p *<* *0.05) based on this two‐way test (similarity of species A vs. B and of species B vs. A), the climatic niches of both species were considered similar, indicating that one species predicts the climatic niche of the other better than would be expected by chance under a specific null model.

## 3 RESULTS

### 3.1 Habitat suitability modeling

Distribution models showed an AUC value above 0.74 for the 10 species of *Xenosaurus*, thus suggesting that the obtained models had high quality and the bioclimatic variables that most contribute to the models’ calibration were Bio7 = Temperature Annual Range, Bio12 = Annual precipitation, and Bio14 = Precipitation of driest month (Table 1).

**Table 1:** Comparison of the top model runs for each species. Values of area under the receiver operating curve (AUC), maximum training sensitivity plus specificity of the models of habitat suitability of the 10 species of the genus *Xenosaurus* are represented. The bioclimatic variables that contributed the most to their construction for each species of *Xenosaurus* were: Bio1 (annual mean temperature), bio7 (annual range temperature), bio11 (annual mean temperature of the coldest quarter), bio12 (annual precipitation), bio 14 (precipitation of the driest month), bio17 (precipitation of the driest quarter), and bio18 (precipitation of the warmest quarter)

Species	Highest AUC current/Maximum test sensitivity plus specificity threshold	Four more important variables and percent of contribution
*Xenosaurus agrenon*	0.79/0.7	Bio 7(44.2), bio 12(35), bio 14(13.8), bio 1(6)
*Xenosaurus grandis*	0.93/0.3	Bio 12(41.7), bio 14(27), bio 7(9.4), bio 11(9.2)
*Xenosaurus mendozai*	0.96/0.09	Bio 14(47.5), bio 7(31.2), bio 12(20.1), bio 11(0.5)
*Xenosaurus newmanorum*	0.91/0.8	Bio 14(65.1), bio 18(22.9), bio 7(11.9), bio 17 (2.3)
*Xenosaurus platyceps*	0.94/0.7	Bio 14(36.7), bio 7(29.8), bio 18(13.5), bio 17(13.3)
*Xenosaurus phalaroanthereon*	0.86/0.5	Bio 7(75.3), bio 17(15.2), bio 12(4.3), bio 1(4.1)
*Xenosaurus rackhami*	0.88/0.4	Bio 7(67.4), bio 12(19.3), bio 14(7.2), bio 18(2.3)
*Xenosaurus rectocollaris*	0.74/0.5	Bio 7(36.9), bio 1(35.3), bio 12(25.3), bio 17(2.5)
*Xenosaurus sanmartinensis*	0.95/0.9	Bio 14(42.5), bio 7(34), bio 12(21.2), bio 11(2.3)
*Xenosaurus tzacualtipantecus*	0.83/0.8	Bio 14(80.8), bio 11(18.7), bio 18(1), bio 17(0.5)

**Figure 3:**
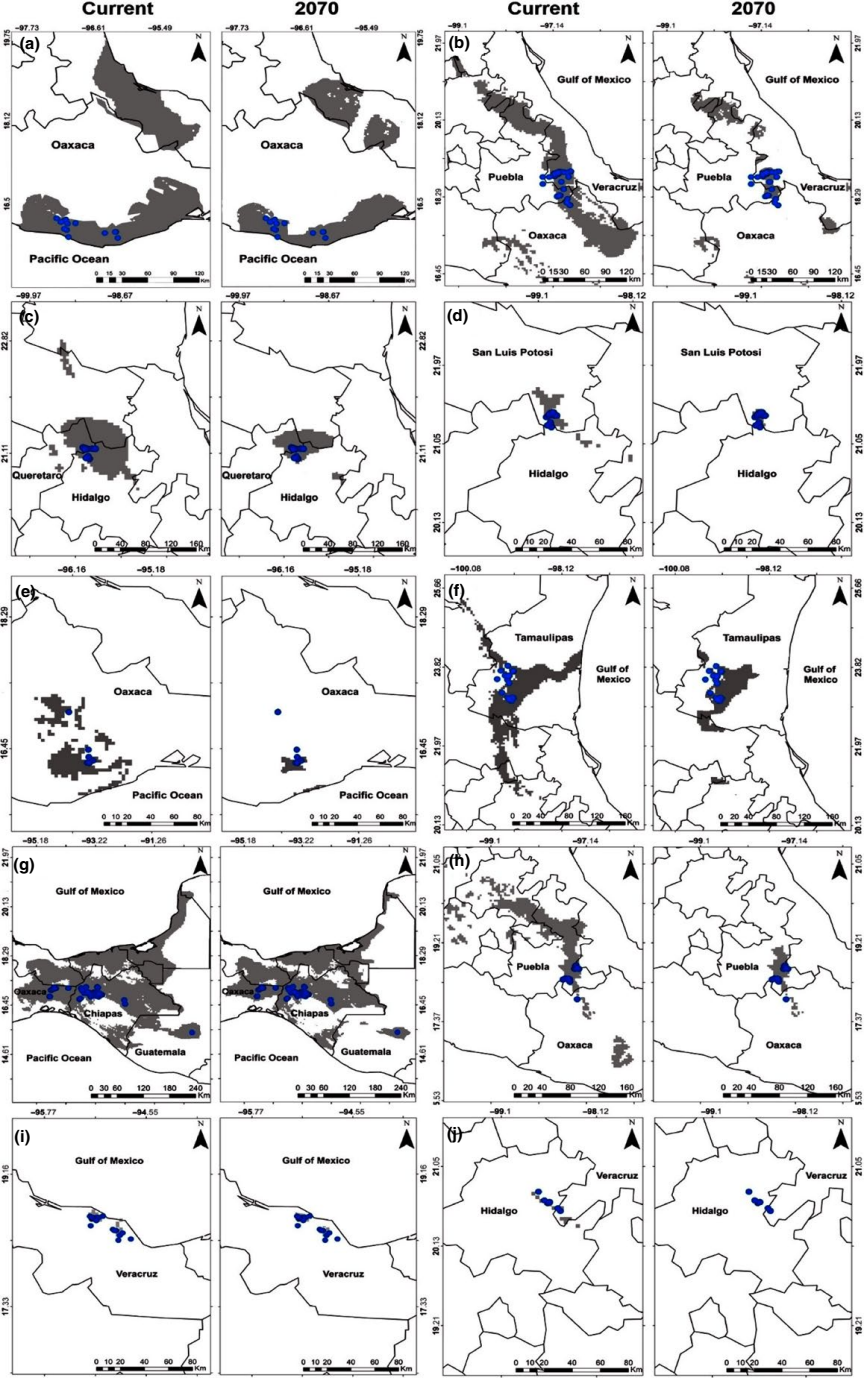
Habitat suitability area from models projected in current and future (2070 RCP85) climatic conditions. Gray color areas represent the climatic niche projected for the species (a) *Xenosaurus agrenon*, (b) *X. grandis*, (c) *X. mendozai*, (d) *X. newmanorum*, (e) *X. phalaroanthereon*, (f) *X. platyceps*, (g) *X. rackhami*, (h) *X. rectocollaris*, (i) *X. sanmartinensis,* and (j) *X. tzacualtipantecus*

Considering a scenario of climatic niche conservatism, all *Xenosaurus* species showed a decrease in their areas of habitat suitability in the future. *Xenosaurus tzacualtipantecus* showed the smallest suitable area (55.13 km^2^), whereas the model obtained for *X*. *rackhami* had the largest suitable area (16,203.11 km^2^) under current climatic conditions. On the other hand, the projected models for the year 2070 showed a 100% area loss for *X*. *tzacualtipantecus*, whereas *X*. *rackhami* maintained the largest suitable area (11,333.18 km^2^) (Table 2; Figure 3).

**Table 2:** Area with suitable climatic conditions in the present and projected future and rate of change of habitat suitability

Species	Current suitable area (km^2^)	2070 projected area (km^2^)	Change rate (%)
*Xenosaurus agrenon*	8,356.33	6,754.87	−19.17
*Xenosaurus grandis*	10,693.59	2,654.21	−75.18
*Xenosaurus mendozai*	13,539.78	6,444.62	−52.40
*Xenosaurus newmanorum*	70.10	40.67	−41.99
*Xenosaurus phalaroanthereon*	75.65	10.78	−85.75
*Xenosaurus platyceps*	2,168.36	1,601.22	−26.15
*Xenosaurus rackhami*	16,203.11	11,333.18	−30.6
*Xenosaurus rectocollaris*	1,102.33	402.59	−63.47
*Xenosaurus sanmartinensis*	134.29	75.63	−43.68
*Xenosaurus tzacualtipantecus*	55.13	0	−100

### 3.2 Similarity of climatic niche (conservatism)

**Figure 4:**
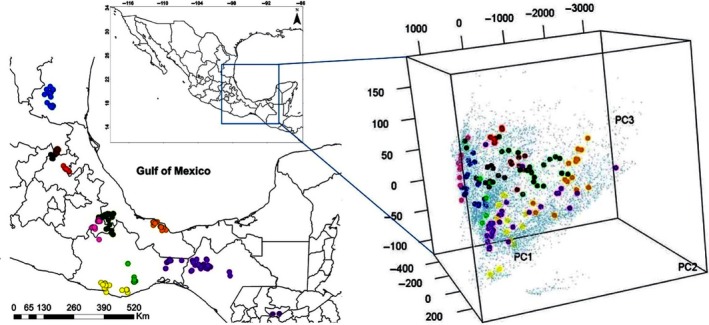
Distribution of the genus *Xenosaurus* in the PCA of climatic space**.** The figure shows the environmental space of *Xenosaurus agrenon* (yellow dots), *X. grandis* (dark green dots), *X. mendozai* (black dots), *X. newmanorum* (brown dots), *X. phalaroanthereon* (light green dots), *X. platyceps* (sharp blue dots), *X. rackhami* (purple dots), *X. rectocollaris* (pink dots), *X. sanmartinensis* (orange dots), and *X. tzacualtipantecus* (red dots). Small dots in blue color show the climatic space

The results of the analyses of climatic niche similarity are presented in Table 3. In general, the values of climatic niche similarity among *Xenosaurus* species were low, suggesting low niche conservatism in this genus. Only the following species pairs showed climatic niche similarity (*p *≤* *0.05): *X*. *grandis*‐*X*. *platyceps*,* X*. *mendozai*‐*X*. *platyceps*,* X*. *platyceps*‐*X*. *rackhami*,* X*. *phalaroanthereon*‐*X*. *rackhami*, and *X*. *rackhami*‐*X*. *sanmartinensis*. Finally, *X*. *agrenon*,* X*. *rectocollaris*,* X*. *newmanorum*, and *X*. *tzacualtipantecus* were not significantly similar to any of the other nine species of the genus (Table 3; Figure 4).

**Table 3:** Similarity indices (overlap) of the climatic niche of the species (*D*) and significance (*PD*). Species abbreviations are: *Xenosaurus agrenon* (sp1), *X*. *grandis* (sp2), *X*. *mendozai* (sp3), *X. newmanorum* (sp4), *X. platyceps* (sp5), *X. phalaroanthereon* (sp6), *X. rackhami* (sp7), *X. rectocollaris* (sp8), *X. sanmartinensis* (sp9), and *X. tzacualtipantecus* (sp10)

Species	Sp2	Sp3	Sp4	Sp5	Sp6	Sp7	Sp8	Sp9	Sp10
Sp1	*D *=* *0 *PD *=* *1–1	*D *=* *0 *PD *=* *1–1	*D *=* *0 *PD *=* *1–1	*D *=* *0 *PD *=* *1–1	*D *=* *0 *PD *=* *1–1	*D *=* *0.217 *PD *=* *0.207–0.009	*D *=* *0 *PD *=* *1–1	*D *=* *0 *PD *=* *1–1	*D *=* *0 *PD *=* *1–1
Sp2		*D *=* *0 *PD *=* *1–1	*D *=* *0 *PD *=* *1–1	*D *=* *0.019 *PD *=* *0.019–0.019^a^	*D *=* *0 *PD *=* *1–1	*D *=* *0.172 *PD *=* *0.772–0.029	*D *=* *0.060 *PD *=* *0.009–0.217	*D *=* *0.127 *PD *=* *0.009–0.485	*D *=* *0 *PD *=* *1–1
Sp3			*D *=* *0 *PD *=* *1–1	*D *=* *0.229 *PD *=* *0.029–0.029^a^	*D *=* *0 *PD *=* *1–1	*D *=* *0.042 *PD *=* *0.475–0.009	*D *=* *0 *PD *=* *1–1	*D *=* *0 *PD *=* *1–1	*D *=* *0 *PD *=* *1–1
Sp4				*D *=* *0 *PD *=* *1–1	*D *=* *0 *PD *=* *1–1	*D *=* *0.022 *PD *=* *0.732–0.009	*D *=* *0 *PD *=* *1–1	*D *=* *0 *PD *=* *1–1	*D *=* *0 *PD *=* *1–1
Sp5					*D *=* *0 *PD *=* *1–1	*D *=* *0.068 *PD *=* *0.772–0.009	*D *=* *0 *PD *=* *1–1	*D *=* *0 *PD *=* *1–1	*D *=* *0 *PD *=* *1–1
Sp6						*D *=* *0.218 *PD *=* *0.009–0.019^a^	*D *=* *0 *PD *=* *1–1	*D *=* *0 *PD *=* *1–1	*D *=* *0 *PD *=* *1–1
Sp7							*D *=* *0.013 *PD *=* *0.72–0.01	*D *=* *0.349 *PD *=* *0.009–0.019^a^	*D *=* *0 *PD *=* *1–1
Sp8								*D *=* *0 *PD *=* *1–1	*D *=* *0 *PD *=* *1–1
Sp9									*D *=* *0 *PD *=* *1–1
Sp10									*0*

Note

a Climatic similarity.

## 4 DISCUSSION

Most of the *Xenosaurus* species studied in this research showed a decrease in habitat suitability in the future considering a climatic niche conservatism scenario (Díaz de la Vega‐Pérez et al., 2013; Rocha & Vrcibradic, 1996). Thus, it would be expected that the species of lizards of this genus should be vulnerable to climate change because this will modify the environmental parameters of the climatic niche where the species occur (Gabriel, Robock, Xia, Zambri, & Kravitz, 2017). For example, the results of the niche models for species *X*. *grandis*,* X*. *mendozai*,* X*. *phalaroanthereon*,* X*. *rectocollaris*, and *X*. *tzacualtipantecus*, which inhabit temperate and cold ecosystems, showed a dramatic decrease in their habitat suitability by 2070. Similar patterns have been reported by Espinosa and Ocegueda (2008) and Villers‐Ruiz and Trejo‐Vázquez (1997), who mentioned that the distribution range of vegetation of ecosystems with Nearctic affinities (associated with cold climates) will decrease due to climatic change. Therefore, on the basis of the aforementioned evidence, it is reasonable to infer that the genus *Xenosaurus* will be exposed to a high vulnerability risk due to climatic change. In this sense, Lemos‐Espinal et al. (2003a) pointed out that *X*. *grandis* might persist in croplands that provide the minimum requirements of microhabitat and native vegetation cover. However, the areas predicted by its habitat suitability model in the future, include croplands and urban settlements, which reduce the possibility of occupying these sites due to the particular ecological characteristics of this species, such as high microhabitat specificity and low dispersion capacity, and therefore, the future survival of its populations will be at high risk (King & Thompson, 1968).

On the other hand, habitat suitability area for species with tropical affinities (tropical montane cloud forests), such as *X*. *agrenon*,* X*. *newmanorum*,* X*. *platyceps*,* X*. *rackhami*, and *X*. *sanmartinensis* (Lemos‐Espinal, Smith, & Woolrich‐Piña, 2012), diminishes to a lesser extent than that of species of temperate and cold environments. Important portions of the climatic niche of these, however, are lost. For example, for the species *X. newmanorum*, the suitable habitat to live in the north of the state of Hidalgo will disappear. An analysis of potential future ecosystem distributions in South America suggests that large extensions of Amazonian rainforest could be replaced by tropical savannahs due to the increase in temperature and decreased rainfall (Lapola, Oyama, & Nobre, 2009). Even if environmental conditions were maintained in the future, changes in vegetation structure could affect these tropical species negatively (Turner, 1996).

According to the variables that contributed the most to the climatic niche models (see Table 1), it is evident that precipitation regime is very important for *Xenosaurus* lizards; also, our results indicate that a decline in precipitation conditions for this species in the area they inhabit is expected under all scenarios, causing more hostile environments for the lizards. For example, Lavergne, Mouquet, Thuiller, and Ronce (2010) noted that organisms might not be able to adapt to climate change if the rate of change is too rapid and the demography is not sufficiently dynamic. Therefore, one of the crucial questions in the debate on ecological effects of climate change is whether species will be able to adapt rapidly enough to keep up with the rapid pace of changing climate (Parmesan, 2006; Wiens et al., 2010).

Our results show that members of the genus *Xenosaurus* have a low similarity among their climatic niches, suggesting low niche conservatism and a tendency to niche shift. Such patterns have been found already in other animal species (Pyron & Burbrink, 2009; Rodrigues, Pacheco Coelho, & Diniz‐Filho, 2016; Strubbe, Beauchard, & Matthysen, 2015). This also might explain the high environmental variability reported for most of the species of *Xenosaurus* (King & Thompson, 1968; Nieto‐Montes de Oca, Campbell, & Flores‐Villela, 2001). Besides, according to the lack of conservatism, *Xenosaurus* species presenting strong reductions in their habitat suitability area in the future, such as *X*. *phalaroanthereon*,* X*. *rectocollaris*, and *X*. *tzacualtipantecus,* might not have the ability to successfully adapt quickly to different conditions imposed by climatic change (Wiens et al., 2010).

The similarity of the climatic niche found in this study does not show a grouping pattern similar to those reported in the recent phylogeny of the *Xenosaurus* lineage proposed by Nieto‐Montes de Oca et al. (2017). Therefore, these results might represent clear evidence that lizard species of *Xenosaurus* do not conserve their ancestral climatic niche. For instance, our results showed that there is only similarity of the climatic niche among the species *X. grandis*,* X. mendozai*,* X. phalaroanthereon*,* X. platyceps*,* X. rackhami*, and *X. sanmartinensis*. If we compare our results with the recent phylogeny, however, only the species *X. platyceps* and *X. mendozai* that belong to the most northern clade (clade *newmanorum*), and the species *X*. *rackhami* and *X*. *sanmartinensis* of the most southern clade (clade *rackhami*) present niche similarity among closely related species, reflecting the little similarity of the climatic niche among the species in this study. Therefore, our findings could be interpreted in light of the group's evolutionary history, considering that *Xenosaurus* taxa are found in independent evolutionary trajectories (Nieto‐Montes de Oca et al., 2017). For example, each species presents different strategies and thermal needs that could also be promoting the variation of life histories presented by each of the species, determined in turn by local adaptations that characterize the populations (Zúñiga‐Vega et al., 2017).

Even the lack of niche conservatism, might not be enough for *Xenosaurus* to successfully adapt in the future. For example, there is evidence that most of the species of *Xenosaurus* need a dense vegetation cover to survive and reproduce. Authors such as Nieto‐Montes de Oca (1999), Lemos‐Espinal and Smith (2005), and Lara‐Tufiño, Ramírez‐Bautista, Hernández‐Austria, Wilson, and Berriozabal‐Islas (2013) pointed out that native vegetation cover provides suitable conditions of humidity and temperature for thermoregulation; therefore, these factors are necessary for the persistence of the species. Thus, if vegetation is disturbed by anthropogenic activities or by climate change, *Xenosaurus* species might lose most of their needed micro‐climatic conditions.

In conclusion, we found that *Xenosaurus* species will face strong reductions in their suitable climatic area due to climate change and that the low values obtained from the indices of climatic similarity among the *Xenosaurus* species suggest that they could respond to local environmental changes. The persistence of the species of Xenosaurus, depends on several factors, such as speed of climate change, accessibility of areas to migrate, vegetation structure, dispersion rate, thermal tolerances, and the change in dimensions of the biotic niche (e.g., new predators and competitors; Loarie et al., 2009). Then, even our optimistic finding of niche shift might not be enough for these species to prevail. For example, species of *Xenosaurus* need micro‐environmental conditions for survival and reproduction (Ballinger et al., 1995; Lemos‐Espinal & Smith, 2005). Therefore, our models could have biases in their accuracy, because these models only consider environmental variables at macro‐scale levels and do not take into account micro‐environmental conditions and interactions (Mateo, Felicísimo, & Muñoz, 2011). Therefore, we propose to do more fine‐scale studies of ethology and thermal ecology (López‐Alcaide, Nakamura, Macip‐Ríos, & Martínez‐Meyer, 2014) in order to establish greater precision in our understanding of the response of these species to increase in the ambient temperature.

Finally, in order to cushion the effects of global climate change, we propose to maintain the remnants of native vegetation, as well as rocky outcrops where *Xenosaurus* populations live, in order to maintain specific habitat conditions. In addition, the conservation status of these lizards must be carefully reviewed in order to fulfill the central obligations of species conservation, particularly for such a vulnerable group.

## CONFLICT OF INTEREST

None declared.

## AUTHOR CONTRIBUTIONS

C.B.I., J.F.M.R., A.R.B, and J.L.B.L. conceived the study; C.B.I., A.R.B, and A.N.M. collated the data, and C.B.I. analyzed the data. All authors contributed to the writing, led by C.B.I.

## DATA ACCESSIBILITY

The location data for the lizards are confidential, because the populations are at severe risk of extinction, due to factors of excessive scientific collection and traffic of species. If the data are required, one can contact C.B.I and A.R.B and we will provide the data.

## References

[ece34200-bib-0001] Ackerly, D. D. , & Reich, P. B. (1999). Convergence and correlations among leaf size and function in seed plants: A comparative test using independent contrasts. American Journal Botany, 86, 1272–1281. 10.2307/2656775 10487815

[ece34200-bib-0002] Adolph, S. C. (1990). Influence of behavioral thermoregulation on microhabitat use by two *Sceloporus* lizards. Ecology, 71, 315–327. 10.2307/1940271

[ece34200-bib-0003] Albon, S. D. , Irvine, R. J. , Halvorsen, O. , Langvatn, R. , Loe, L. , Ropstad, E. , … Lee, A. M. (2017). Contrasting effects of summer and winter warming on body mass explain population dynamics in a food‐limited Arctic herbivore. Global Change Biology, 23, 1374–1389. 10.1111/gcb.13435 27426229

[ece34200-bib-0004] Andrews, R. M. (1998). Geographic variation in field body temperature of *Sceloporus* lizards. Journal of Thermal Biology, 23, 329–334. 10.1016/S0306-4565(98)00018-7

[ece34200-bib-0005] Ballesteros‐Barrera, C. , Martínez‐Meyer, E. , & Gadsden, H. (2007). Effects of land‐cover transformation and climate change on the distribution of two microendemic lizards, genus *Uma*, of northern Mexico. Journal of Herpetology, 41, 733–740. 10.1670/06-276.1

[ece34200-bib-0006] Ballinger, R. E. , Lemos‐Espinal, J. A. , Sanoja‐Sarabia, S. , & Coady, N. R. (1995). Ecological observations of the lizard, *Xenosaurus grandis* in Cuautlapan, Veracruz, Mexico. Biotropica, 27, 128–132. 10.2307/2388910

[ece34200-bib-0007] Ballinger, R. E. , Lemos‐Espinal, J. A. , & Smith, G. R. (2000). Reproduction in females of three species of crevice‐dwelling lizards (genus *Xenosaurus*) from Mexico. Studies on Neotropical Fauna and Environment, 35, 179–183. 10.1076/snfe.35.3.179.8857

[ece34200-bib-0008] Bhullar, B. A. S. (2011). The power and utility of morphological characters in systematics: A fully resolved phylogeny of *Xenosaurus* and its fossil relatives (Squamata: Anguimorpha). Bulletin of the Museum of Comparative Zoology, 160, 65–181. 10.3099/0027-4100-160.3.65

[ece34200-bib-0009] Bogert, C. M. (1949). Thermoregulation and eccritic body temperatures in Mexican lizards of the genus *Sceloporus* . Anales del Instituto de Biología, México, 20, 415–426.

[ece34200-bib-0010] Bonetti, M. F. , & Wiens, J. J. (2014). Evolution of climatic niche specialization: A phylogenetic analysis in amphibians. Proceedings of the Royal Society B, 281, 1–9. 10.1098/rspb.2013.3229 PMC4213603 25274369

[ece34200-bib-0011] Broennimann, O. , Fitzpatrick, M. C. , Pearman, P. B. , Petitpierre, B. , Pellissier, L. , & Yoccoz, N. G. (2012). Measuring ecological niche overlap from occurrence and spatial environmental data. Global Ecology and Biogeography, 21, 481–497. 10.1111/j.1466-8238.2011.00698.x

[ece34200-bib-0012] Broennimann, O. , Petitpierre, B. , Randin, C. , Engler, R. , Cola, V. D. , Breiner, F. , … Guisan, A. (2015). Ecospat: Spatial ecology miscellaneous methods. R package version 1.1. Retrieved from http://CRAN.R-project.org/package=ecospat.

[ece34200-bib-0013] Burgman, M. A. , & Fox, J. C. (2003). Bias in species range estimates from minimum convex polygons: Implications for conservation and options for improved planning. Animal Conservation, 6, 19–28. 10.1017/S1367943003003044

[ece34200-bib-0014] Cianfrani, C. , Lay, G. L. , Hirzel, A. H. , & Loy, A. (2010). Do habitat suitability models reliably predict the recovery areas of threatened species? Journal of Applied Ecology, 47, 421–430. 10.1111/(ISSN)1365-2664

[ece34200-bib-0015] Deutsch, C. A. , Tewksbury, J. J. , Huey, R. B. , Sheldon, K. S. , Ghalambor, C. K. , Haak, D. C. , & Martin, P. R. (2008). Impacts of climate warming on terrestrial ectotherms across latitude. Proceedings of the National Academy of the United States of America, 18, 6668–6672. 10.1073/pnas.0709472105 PMC2373333 18458348

[ece34200-bib-0016] Di Cola, V. , Broennimann, O. , Petitpierre, B. , Breiner, F. T. , D'Amen, M. , Randin, C. , … Guisan, A. (2017). Ecospat: An R package to support spatial analyses and modeling of species niches and distributions. Ecography, 40, 774–787. 10.1111/ecog.02671

[ece34200-bib-0017] Díaz de la Vega‐Pérez, A. H. , Jiménez‐Arcos, V. H. , Manríquez‐Morán, N. L. , & Méndez‐De la Cruz, F. R. (2013). Conservatism of thermal preferences between parthenogenetic *Aspidoscelis cozumela* complex (Squamata: Teiidae) and their parental species. Herpetological Journal, 23, 93–104.

[ece34200-bib-0018] Elith, J. , Graham, C. H. , Anderson, R. P. , Dudík, M. , Ferrier, S. , Guisan, A. , … Zimmermann, N. E. (2006). Novel methods improve prediction of species’ distributions from occurrence data. Ecography, 29, 129–151. 10.1111/j.2006.0906-7590.04596.x

[ece34200-bib-0019] Elith, J. , Kearney, M. , & Phillips, S. (2010). The art of modelling range‐shifting species. Methods in Ecology and Evolution, 1, 330–342. 10.1111/j.2041-210X.2010.00036.x

[ece34200-bib-0020] Elith, J. , Phillips, S. J. , Hastie, T. , Dudík, M. , Chee, Y. E. , & Yates, C. J. (2011). A statistical explanation of MaxEnt for ecologists. Diversity and Distributions, 17, 43–57. 10.1111/j.1472-4642.2010.00725.x

[ece34200-bib-0021] Espinosa, D. , & Ocegueda, S. (2008). El conocimiento biogeográfico de las especies y su regionalización natural In Soberón J., Halffter G., & Llorente‐Bousquets J. (Eds.), Conocimiento actual de la biodiversidad, Vol. 1 (pp. 33–65). México: Comisión Nacional para el Conocimiento y Uso de la Biodiversidad.

[ece34200-bib-0022] ESRI (2014). ArcGIS 10.3. Redlands, CA: Environmental Systems Research Institute

[ece34200-bib-0023] Gabriel, C. J. , Robock, A. , Xia, L. , Zambri, B. , & Kravitz, B. (2017). The G4Foam Experiment: Global climate impacts of regional ocean albedo modification. Atmospheric Chemistry and Physics, 17, 595–613. 10.5194/acp-17-595-2017

[ece34200-bib-0024] García, A. , Ortega‐Huerta, M. A. , & Martínez‐Meyer, E. (2013). Potential distributional changes and conservation priorities of endemic amphibians in western Mexico as a result of climate change. Environmental Conservation, 41, 1–12.

[ece34200-bib-0025] García‐Rico, J. , Díaz de la Vega‐Pérez, A. , Smith, G. R. , Lemos‐Espinal, J. A. , & Woolrich‐Piña, G. A. (2015). Thermal ecology, sexual dimorphism, and diet of *Xenosaurus tzacualtipantecus* from Hidalgo, Mexico. Western North American Naturalist, 75, 209–217. 10.3398/064.075.0209

[ece34200-bib-0027] Grigg, J. W. , & Buckley, L. B. (2013). Conservatism of lizard thermal tolerances and body temperatures across evolutionary history and geography. Biology Letters, 9, 20121056 10.1098/rsbl.2012.1056 23325735 PMC3639756

[ece34200-bib-0028] Gutiérrez, E. , & Trejo, I. (2014). Efecto del cambio climático en la distribución potencial de cinco especies arbóreas de bosque templado en México. Revista Mexicana de Biodiversidad, 85, 179–188. 10.7550/rmb.37737

[ece34200-bib-0029] Hijmans, R. J. , Cameron, S. E. , Parra, J. L. , Jones, P. G. , & Jarvis, A. (2005). Very high resolution interpolated climate surfaces for global land areas. International Journal of Climatology, 25, 1965–1978. 10.1002/(ISSN)1097-0088

[ece34200-bib-0031] Hu, J. , Broennimann, O. , Guisan, A. , Wang, B. , Huang, Y. , & Jianping, J. (2016). Niche conservatism in *Gynandropaa* frogs on the southeastern Qinghai‐Tibetan Plateau. Scientific Reports, 6, 32624 10.1038/srep32624 27601098 PMC5013482

[ece34200-bib-0032] Huey, R. B. , & Kingsolver, J. G. (1993). Evolution of resistance to high temperature in ectotherms. The American Naturalist, 142, 21–46. 10.1086/285521

[ece34200-bib-0033] Jackson, M. M. , Gergel, S. E. , & Martin, K. (2015). Effects of climate change on habitat availability and configuration for an endemic coastal alpine bird. PLoS ONE, 10, 0142110. 10.1371/journal.pone.0142110 PMC4631505 26529306

[ece34200-bib-0034] King, W. , & Thompson, F. G. (1968). A review of the American lizards of the genus *Xenosaurus* Peters. Bulletin of the Florida State Museum, 12, 93–123.

[ece34200-bib-0035] Lapola, D. M. , Oyama, M. D. , & Nobre, C. A. (2009). Exploring the range of climate biome projections for tropical South America: The role of CO_2_ fertilization and seasonality. Global Biogeochemical Cycles, 23, 93–123. 10.1029/2008GB003357

[ece34200-bib-0036] Lara‐Tufiño, D. , Ramírez‐Bautista, A. , Hernández‐Austria, R. , Wilson, L. D. , & Berriozabal‐Islas, C. (2013). *Xenosaurus newmanorum* Taylor, 1949 (Squamata: Xenosauridae): Occurrence in the state of Hidalgo, Mexico. Check List, 9, 1101–1103. 10.15560/9.5.1101

[ece34200-bib-0037] Lavergne, S. , Mouquet, N. , Thuiller, W. , & Ronce, O. (2010). Biodiversity and climate change: Integrating evolutionary and ecological responses of species and communities. Annual Review of Ecology and Evolutionary Systematics, 41, 321–350. 10.1146/annurev-ecolsys-102209-144628

[ece34200-bib-0039] Lemos‐Espinal, J. A. , & Smith, G. R. (2005). Natural history of *Xenosaurus phalaroanthereon* (Squamata, Xenosauridae), a knob‐scale lizard from Oaxaca, Mexico. Phyllomedusa, 4, 133–137. 10.11606/issn.2316-9079.v4i2p133-137

[ece34200-bib-0040] Lemos‐Espinal, J. A. , Smith, G. R. , & Ballinger, R. E. (2003a). Ecology of *Xenosaurus grandis agrenon*, a knob‐scaled lizard from Oaxaca, Mexico. Journal of Herpetology, 37, 192–196. 10.1670/0022-1511(2003)037[0192:EOXGAA]2.0.CO;2

[ece34200-bib-0041] Lemos‐Espinal, J. A. , Smith, G. R. , & Ballinger, R. E. (2003b). Variation in growth and demography of a knob‐scaled lizard (*Xenosaurus newmanorum*: Xenosauridae) from a seasonal tropical environment in México. Biotropica, 35, 240–249.

[ece34200-bib-0042] Lemos‐Espinal, J. A. , Smith, G. R. , & Woolrich‐Piña, G. A. (2012). The family Xenosauridae in Mexico, 1st ed. China: ECO Herpetological publishing & distribution.

[ece34200-bib-0043] Liu, X. , Petitpierre, B. , Broennimann, O. , Li, X. , Guisan, A. , & Li, Y. (2017). Realized climatic niches are conserved along maximum temperatures among herpetofaunal invaders. Journal of Biogeography, 44, 111–121. 10.1111/jbi.12808

[ece34200-bib-0044] Loarie, S. R. , Duffy, P. B. , Hamilton, H. , Asner, G. P. , Field, C. B. , & Ackerly, D. D. (2009). The velocity of climatic change. Nature, 462, 1052–1055. 10.1038/nature08649 20033047

[ece34200-bib-0045] Lobo, J. M. , Jiménez‐Valverde, A. , & Real, R. (2007). AUC: A misleading measure of the performance of predictive distribution models. Global Ecology and Biogeography, 17, 145–151.

[ece34200-bib-0046] López‐Alcaide, S. , Nakamura, M. , Macip‐Ríos, R. , & Martínez‐Meyer, E. (2014). Does behavioural thermoregulation help pregnant *Sceloporus adleri* lizards in dealing with fast environmental temperature rise? Herpetological Journal, 24, 41–47.

[ece34200-bib-0047] Macey, J. R. , Schulte, J. A. II , Larson, A. , Tuniyev, B. S. , Orlov, N. , & Papenfuss, T. J. (1999). Molecular phylogenetics, tRNA evolution, and historical biogeography in Anguid lizards and related taxonomic families. Molecular Phylogenetics and Evolution, 12, 250–272. 10.1006/mpev.1999.0615 10413621

[ece34200-bib-0048] Malcolm, J. R. , Liu, C. , Neilson, R. P. , Hansen, L. , & Hannah, L. (2006). Global warming and extinctions of endemic species from biodiversity hotspots. Conservation Biology, 20, 538–548. 10.1111/j.1523-1739.2006.00364.x 16903114

[ece34200-bib-0049] Mateo, R. G. , Felicísimo, Á. M. , & Muñoz, J. (2011). Modelos de distribución de especies: Una revisión sintética. Revista Chilena de Historia Natura, 84, 217–240. 10.4067/S0716-078X2011000200008

[ece34200-bib-0050] Meinshausen, M. , Smith, S. J. , Calvin, K. , Daniel, J. S. , Kainuma, M. , Lamarque, J. F. , … van Vuuren, D. P. (2011). The RCP greenhouse gas concentrations and their extensions from 1765 to 2300. Climatic Change, 109, 213–241. 10.1007/s10584-011-0156-z

[ece34200-bib-0051] Menezes, V. A. , & Rocha, C. F. D. (2011). Thermal ecology *Cnemidophorus* species (Squamata: Teiidae) in east of Brazil. Journal of Thermal Biology, 10, 10–16.

[ece34200-bib-0052] Merow, C. , Smith, M. J. , & Silander, J. A. (2013). A practical guide to MaxEnt for modeling species’ distributions: What it does, and why inputs and settings matter. Ecography, 36, 1058–1069. 10.1111/j.1600-0587.2013.07872.x

[ece34200-bib-0053] Moreno‐Amat, E. , Mateo, R. G. , Nieto‐Lugilde, D. , Morueta‐Holme, N. , Svenning, J. C. , & García‐Amorena, I. (2015). Impact of model complexity on cross‐temporal transferability in Maxent species distribution models: An assessment using paleobotanical data. Ecological Modelling, 312, 308–317. 10.1016/j.ecolmodel.2015.05.035

[ece34200-bib-0054] Nieto‐Montes de Oca, A. (1999). Sistemática y biogeografía del género Xenosaurus (Squamata: Xenosauridae). Universidad Nacional Autónoma de México Facultad de Ciencias Departamento de Biología Museo de Zoología “Alfonso L Herrera”. Reporte final No.: SNIB‐CONABIO. Contract No.: H245.

[ece34200-bib-0055] Nieto‐Montes de Oca, A. , Barley, A. J. , Meza‐Lázaro, R. N. , García‐Vázquez, U. O. , Zamora‐Abrego, J. G. , Thomson, R. C. , & Leaché, A. D. (2017). Phylogenomics and species delimitation in the knob‐scaled lizards of the genus *Xenosaurus* (Squamata: Xenosauridae) using ddRADseq data reveal a substantial underestimation of diversity. Molecular Phylogenetics and Evolution, 106, 241–253. 10.1016/j.ympev.2016.09.001 27720785

[ece34200-bib-0056] Nieto‐Montes de Oca, A. , Campbell, J. A. , & Flores‐Villela, O. (2001). A new species of *Xenosaurus* (Squamata: Xenosauridae) from the Sierra Madre del Sur of Oaxaca, Mexico. Herpetologica, 57, 32–47.

[ece34200-bib-0057] Nieto‐Montes de Oca, A. , García‐Vázquez, U. O. , Zúñiga‐Vega, J. J. , & Schmidt‐Ballardo, W. (2013). A new species of *Xenosaurus* (Squamata: Xenosauridae) from the Sierra Gorda Biosphere Reserve of Queretaro, Mexico. Revista Mexicana de Biodiversidad, 84, 485–498. 10.7550/rmb.35733

[ece34200-bib-0058] Parmesan, C. (2006). Ecological and evolutionary responses to recent climate change. Annual Review of Ecology, Evolution, and Systematics, 37, 637–669. 10.1146/annurev.ecolsys.37.091305.110100

[ece34200-bib-0060] Phillips, S. J. , Anderson, R. P. , & Schapire, R. E. (2006). Maximum entropy modeling of species geographic distributions. Ecological Modelling, 190, 231–259. 10.1016/j.ecolmodel.2005.03.026

[ece34200-bib-0061] Phillips, S. J. , Dudik, M. , & Schapire, R. E. (2004). A maximum entropy approach to species distribution modeling In Greiner R. & Schuurmans D. (Eds.), Proceedings of the twenty‐first international conference on machine learning (pp. 655–662). New York, NY: Association for Computing.

[ece34200-bib-0062] Pianka, E. R. (1970). Comparative autecology of the lizard *Cnemidophorus tigris* in different parts of its geographic range. Ecology, 51, 703–720. 10.2307/1934053

[ece34200-bib-0063] Pyron, R. A. , & Burbrink, F. T. (2009). Lineage diversification in a widespread species: Roles for niche divergence and conservatism in the common kingsnake. Lampropeltis getula, 18, 3443–3457. 10.1111/j.1365-294X.2009.04292.x 19659478

[ece34200-bib-0064] Rocha, C. F. D. , & Vrcibradic, D. (1996). Thermal ecology of two sympatric skinks (*Mabuya macrorhyncha* and *Mabuya agilis*) in a Brazilian restinga habitat. Australian Journal of Ecology, 21, 110–113. 10.1111/j.1442-9993.1996.tb00590.x

[ece34200-bib-0065] Rodrigues, J. F. M. , Pacheco Coelho, M. T. , & Diniz‐Filho, J. A. F. (2016). Exploring intraspecific climatic niche conservatism to better understand species invasion: The case of *Trachemys dorbigni* (Testudines, Emydidae). Hydrobiologia, 779, 127–134. 10.1007/s10750-016-2805-8

[ece34200-bib-0066] Rojas‐González, R. I. , Jones, C. P. , Zúñiga‐Vega, J. J. , & Lemos‐Espinal, J. A. (2008). Demography of *Xenosaurus platyceps* (Squamata: Xenosauridae): A comparison between tropical and temperate populations. Amphibia‐Reptilia, 29, 245–256. 10.1163/156853808784124992

[ece34200-bib-0067] Schall, J. J. (1977). Thermal ecology of five sympatric species of *Cnemidophorus* (Sauria: Teiidae). Herpetologica, 33, 261–272.

[ece34200-bib-0068] Schoener, T. W. (1970). Nonsynchronous spatial overlap of lizards in patchy habitats. Ecology, 51, 408–418. 10.2307/1935376

[ece34200-bib-0069] Sinervo, B. , Méndez‐de‐la‐Cruz, F. , Miles, D. B. , Heulin, B. , Bastiaans, E. , Villagrán‐Santa, C. M. , … Sites, J. W. Jr (2010). Erosion of lizard diversity by climate change and altered thermal niches. Science, 328, 894–899. 10.1126/science.1184695 20466932

[ece34200-bib-0070] Stevens, T. P. (1982). Body temperatures of montane *Cnemidophorus inornatus* (Reptilia: Teiidae). Southwestern Naturalist, 27, 232–234. 10.2307/3671164

[ece34200-bib-0071] Strubbe, D. , Beauchard, O. , & Matthysen, E. (2015). Niche conservatism among non‐native vertebrates in Europe and North America. Ecography, 38, 321–329. 10.1111/ecog.00632

[ece34200-bib-0072] Thuiller, W. , Midgley, G. F. , Hughes, G. O. , Bomhard, B. , Drew, G. , Rutherford, M. C. , & Woodward, F. I. (2006). Endemic species and ecosystem sensitivity to climate change in Namibia. Global Change Biology, 12, 759–776. 10.1111/j.1365-2486.2006.01140.x

[ece34200-bib-0073] Turner, I. M. (1996). Species loss in fragments of tropical rain forest: A review of the evidence. Journal of Applied Ecology, 33, 200–209. 10.2307/2404743

[ece34200-bib-0074] Valenzuela‐Ceballos, S. , Castañeda‐Gaytán, G. , Rioja‐Paradela, T. , Carrillo‐Reyes, A. , & Bastiaans, E. (2015). Variation in the thermal ecology of an endemic iguana from Mexico reduces its vulnerability to global warming. Journal of Thermal Biology, 48, 56–64. 10.1016/j.jtherbio.2014.12.011 25660631

[ece34200-bib-0075] Varela, S. , Anderson, R. P. , García‐Valdés, R. , & Fernández‐González, F. (2014). Environmental filters reduce the effects of sampling bias and improve predictions of ecological niche models. Ecography, 37, 1084–1091.

[ece34200-bib-0076] Villers‐Ruiz, L. , & Trejo‐Vázquez, I. (1997). Assessment of the vulnerability of forest ecosystems to climate change in Mexico. Climate Research, 9, 87–93. 10.3354/cr009087

[ece34200-bib-0078] Warren, D. L. , Glor, R. E. , & Turelli, M. (2008). Environmental niche equivalency versus conservatism: Quantitative approaches to niche evolution. Evolution, 62, 2868–2883. 10.1111/j.1558-5646.2008.00482.x 18752605

[ece34200-bib-0079] Wiens, J. J. , Ackerly, D. D. , Allen, A. P. , Anacker, B. L. , Buckley, L. B. , Cornell, H. V. , … Stephens, P. R. (2010). Niche conservatism as an emerging principle in ecology and conservation biology. Ecology Letters, 13, 1310–1324. 10.1111/j.1461-0248.2010.01515.x 20649638

[ece34200-bib-0080] Winne, C. T. , & Keck, M. B. (2004). Daily activity patterns of Whiptail Lizards (Squamata: Teiidae: *Aspidoscelis*): A proximate response to environmental conditions or an endogenous rhythm? Functional Ecology, 18, 314–321. 10.1111/j.0269-8463.2004.00819.x

[ece34200-bib-0081] Woolrich‐Piña, G. A. , Lemos‐Espinal, J. A. , Oliver‐López, L. , & Smith, G. R. (2012). Ecology of *Xenosaurus rectocollaris* in Tehuacan Valley, Puebla, Mexico. The Southwestern Naturalist, 57, 157–161. 10.1894/0038-4909-57.2.157

[ece34200-bib-0082] Zamora‐Abrego, J. G. , & Ortega‐León, A. M. (2016). Ecología trófica de la lagartija *Xenosaurus mendozai* (Squamata: Xenosauridae) en el estado de Querétaro, México. Revista Mexicana de Biodiversidad, 87, 140–149. 10.1016/j.rmb.2016.01.011

[ece34200-bib-0083] Zúñiga‐Vega, J. J. , Fuentes‐G, J. A. , Zamora‐Abrego, J. G. , García‐Vázquez, U. O. , Nieto‐Montes de Oca, A. , & Martins, E. P. (2017). Evolutionary patterns in life‐history traits of lizards of the genus *Xenosaurus* . Herpetological Journal, 27, 346–360.

